# A Case Study in Breast Density Evaluation Using Bioimpedance Measurements

**DOI:** 10.3390/s22072747

**Published:** 2022-04-02

**Authors:** Marcos Gutiérrez-Lopez, Juan Prado-Olivarez, Carolina Matheus-Troconis, Alfredo Padilla-Medina, Alejandro I. Barranco-Gutiérrez, Alejandro Espinosa-Calderon, Carlos A. Herrera-Ramírez, Javier Diaz-Carmona

**Affiliations:** 1 Electronics Engineering Department, Tecnológico Nacional de México (TecNM), Campus I, Celaya 38010, Mexico; marcos.gutierrez@itcelaya.edu.mx (M.G.-L.); juan.prado@itcelaya.edu.mx (J.P.-O.); alfredo.padilla@itcelaya.edu.mx (A.P.-M.); israel.barranco@itcelaya.edu.mx (A.I.B.-G.); 2 Centro de Diagnóstico Médico, Celaya 38070, Mexico; caromatheus@hotmail.com; 3 CRODE, Tecnológico Nacional de México (TecNM), Celaya 38023, Mexico; alejandro.espinosa@crodecelaya.edu.mx; 4 Robotics Engineering Department, Universidad Politécnica de Guanajuato, Cortazar 38496, Mexico; aherrera@upgto.edu.mx

**Keywords:** breast density, bioimpedance, breast cancer risk, in vivo evaluation

## Abstract

(1) Background: As breast cancer studies suggest, a high percentage of breast density (PBD) may be related to breast cancer incidence. Although PBD screening is one of the strongest predictors of breast cancer risk, X-ray-based mammography evaluation is subjective. Therefore, new objective PBD measuring techniques are of interest. A case study analyzing the PBD of thirteen female participants using a bioimpedance-based method, the anomalies tracking circle (ATC), is described in this paper. (2) Methods: In the first stage, the breast bioimpedance of each participant was measured. Then, the participant breast density was determined by applying a mammogram just after the breast bioimpedance measurement stage. In the third stage, the ATC algorithm was applied to the measured bioimpedance data for each participant, and a results analysis was done. (3) Results: An ATC variation according to the breast density was observed from the obtained data, this allowed the use of classification techniques to determine the PBD. (4) Conclusions: The described breast density method is a promising approach that might be applied as an auxiliary tool to the mammography in order to obtain precise and objective results for evaluation of breast density and with that determine potential breast cancer risk.

## 1. Introduction

Breast density is a term that describes the relative amount of glandular, connective, and fat tissue, which is evaluated from a mammogram. Dense breasts have relatively high amounts of glandular tissue and fibrous connective tissue and relatively low amounts of fatty breast tissue. Thus, the fibrous and glandular tissue appears whiter in a mammogram because fewer X-rays reach the image detector, and fat areas appear darker because more X-rays reach the detector. The relative amount of white and black determines mammographic density [1].

As breast cancer studies suggest, a high percentage of breast density (PBD) may be related to breast cancer incidence [2,3,4]. Additionally, a higher cancer risk is presented in the next five or ten years after the first mammography breast density evaluation [5]. A high PBD is strongly associated with all breast cancer subtypes, but particularly large tumors and positive lymph nodes at all ages, as well as negative estrogen receptor (ER) among women under 55 years old [6]. As women get older, smaller PBD values are obtained, which apparently contradicts the fact that a greater number of breast cancer incidences happen in older women [5]. On the other hand, a woman’s weight is inversely linked to her PBD value, since a smaller/greater dense breast tissue volume is presented in women having greater/smaller weight [7]. Factors such as high levels of alcohol consumption [8] and saturated fat intake are associated with a woman’s PBD [9], which may cause a prevalence of high dense breast patterns. The PBD variability of 20% up to 30% among women may be due to the aforementioned factors and 60% to the genetic inheritance [10,11,12]. Future applications using PBD include improvements in mammographic screening, risk prediction, breast cancer research, and clinical decision-making [6,13]. One potential application currently being researched is to predict breast cancer risk and to make intervention proposals in order to achieve risk reduction [5]. 

According to the Breast Imaging Data and Reporting System (BI-RADS), a PBD increase along a three-year period is associated with a higher breast cancer risk, and a PBD decrease with a smaller risk, than the risk when PBD remains unchanged. Two longitudinal BI-RADS breast density measurements (on current and previous mammography) may be better predictors of breast cancer risk than a single measurement [14]. The incorporation of breast density to the Gail model, a mathematical instrument that measures the risk of developing breast cancer in five years by identifying risk factors [15], increases predictive accuracy by statistical agreement from 0.607 to 0.642 [16]. The PBD BI-RADS classification is defined in Table 1, where illustrative cranio-caudal (CC) and mid-lateral oblique (MLO) mammography imaging samples for right and left breast are shown.

Although PBD screening is one of the strongest predictors of breast cancer risk, X-ray-based mammography is recommended for women over 40 years old [17]. Therefore, new PBD measuring techniques in young women and adolescents are of interest [18].

The qualitative methods for visual assessment of breast density have the disadvantage of results variability due to the subjective approach. The breast density can be estimated using two-dimensional (2D) mammograms without considering the breast thickness; it is done with the breast projected area rather than its volume. The implied subjective error can be notably reduced using automated volumetric breast measurement software [19], but this kind of tool is not commonly used due to its recent US. Food and Drug Administration approval, which makes the visual assessment technique the most widely applied by medical specialist in mammary evaluation. 

Currently, alternative methods are being developed to improve the breast density measurement. The methods include volumetric measurement of breast density in digital mammography [19], ultrasound tomography [13], magnetic resonance imaging [20], and dual-energy X-ray absorptiometry [21]. Although they are viable alternatives, they are not suitable for large population and longitudinal studies due to the high cost involved in their implementation. The PBD estimation based on breast bioimpedance measurement has been proposed as a solution to this issue. As it is known, the fat bioimpedance value is eight times greater than the breast and stromal tissues [22,23]. Hence, the tissue bioimpedance values of dense breast are much lower than those corresponding to a breast with a large amount of adipose tissue [18]. 

An electric breast densitometer (EBD) was used in the reported pilot study in [18], in which the EBD measurements were compared with mammographic densities for adult women and a measurement feasibility of breast density for young girls was evaluated. The obtained EBD measurements of right and left breast of each participant were averaged, being a total of 95 women (21 from Guam and 74 from Hawaii). The measurement accuracy was affected by the sensor size, since it was originally designed for adult Caucasian women, causing sensor contact issues with the breast skin [24]. A small correlation (*Spearman* = −0.52, *p* < 0.0001) between the EBD and PBD measurements was reported, which stands out the need for further evaluation and EBD device development. 

A case study analyzing the PBD of thirteen female participants using a bioimpedance-based method is described in this paper. The PBD, clinically diagnosed by a specialist in mammary radiology, is compared with the results of applying the ATC) algorithm, which is based on breast bioimpedance measurements for locating breast areas with the minimum bioimpedance and reported for locating carcinoma emulators on breast models [25]. From the analysis of the experimentally obtained results, a new approach using the resulted ATC radius is suggested to objectively evaluate the PBD according to the BI-RADS classification.

## 2. Materials and Methods

Bioimpedance measures the opposition offered by a biological medium (cells, fluids, tissues, or organs) to the passage of an ionic current. If these measurements are made within a certain range of frequencies, electrical impedance spectroscopy is obtained, which makes it possible to analyze the impedance behavior on a biological medium according to its basic electrical properties (resistance, capacitance, and inductance). There are different techniques for measuring electrical impedance, depending on the number of electrodes used. The bipolar technique is very popular due to its simplicity, in which the same electrode pairs are used for voltage and current measurement. In this technique, the electrodes resistance must be considered [26]. In addition, spurious impedance readings may exist, due to a large impedance electrodes polarization [27]. On the other hand, the quadrapolar measuring technique achieves a cleaner measurement by isolating the voltage measurement from the electrode resistance. However, even in a four-electrode system, the current flow through parasitic capacitances and the contact impedances of the electrode-skin system can cause very significant errors and phase changes [26,28].

**Figure 1:**
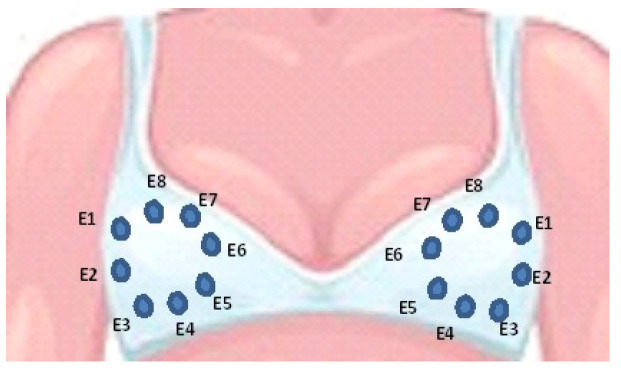
Distribution of the electrodes E1–E8 in the latex cups.

**Figure 2:**
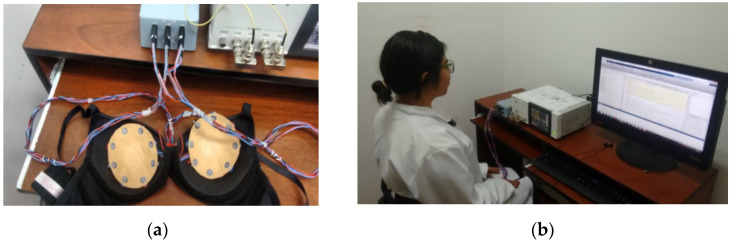
Bioimpedance measurement system: (**a**) designed latex cups within brassiere; (**b**) measuring participant’s breast bioimpedance.

Bioimpedance of both breasts on thirteen voluntary female participants were measured in the first experimental stage. The participants were women in an age range of 40 up to 60 years old, with a B brassier cup size, and none of them had breast lesions or biopsies in their clinical history. All participants were adequately informed about the applied procedure and asked to sign a letter of informed consent. The bioimpedance measurements were done based on the system described in [25] but using an impedance analyzer model HIOKI IM-3536 and a pair of designed latex B size cups with eight Ag/AgCl electrodes distributed in a ring array as illustrated in Figure 1. The designed cups were placed and held on a brassier using Velcro, as shown in Figure 2a. The measuring procedure consisted of first correctly putting the brassier on the participant, who was seated front and near to the analyzer, as illustrated in Figure 2b. All the bioimpedance measurements were done with a constant voltage of 0.2 volts and a limited electrical current up to 60 micro-amperes, which are safe values for applying on human beings according to the IEC/TS 60479-1 standard [29]. The breast bioimpedances were systematically measured for the electrode ring array in each breast following the procedure described in [25]. A total of 112 measured data values at a frequency of 100 KHz was obtained for each participant’s breast with an approximately measuring time of 11 s.

In the second experimental stage, the breast densities for both breasts in each participant were determined. This was done by applying a mammogram just after the breast bioimpedance measurement stage. From the CC and MLO mammograms image, for the right (R) and left (L) breasts, a specialist with twenty-two years of experience in mammary radiology determined the PBD of each participant using the BI-RADS classification. The BI-RADS PBD evaluated by the mammary radiology specialist for each participant is shown in Table 2.

In the third experimental stage, the ATC algorithm [25] was applied to the measured bioimpedance data for each participant. The lowest impedance area within the domain of interest was determined by the centroid of the interception points of eight lines representing the lowest bioimpedance measurements within a subset of seven impedance measurements.

## 3. Results

The obtained ATC results and the corresponding mammogram images for each participant, right (R) and left (L) breast, are depicted in Table 3. The white dot in left image represents a visually identification of the nipple physically location on the mammography image An ATC variation according to the breast density can be observed from the obtained results.

**Figure 3:**
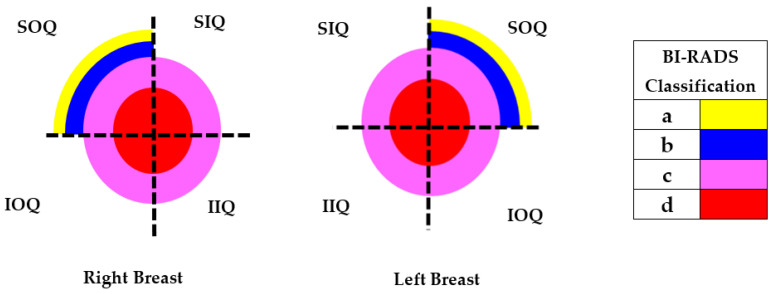
Proposed ATC concentric zones according to the BI-RADS classification.

The breast zones are defined in quadrants and named from a frontal view of each breast: superior outer quadrant (SOQ), superior inner quadrant (SIQ), inferior outer quadrant (IOQ) and inferior inner quadrant (IIQ). A trend of the ATC results with respect to the BI-RADS PBD was observed after analyzing the obtained ATC data for each participant. Most of the resulting positions of the ATC algorithm for low PBD values (BI-RADS b) are located on the SOQ, while greater PBD values (BI-RADS c and d) resulted on positions near to the center, regardless of the quadrant. Hence, a concentric zones distribution of the resulted ATC positions according to the BI-RADS classification is proposed and graphically depicted in Figure 3.

The resulting ATC positions for all participants were subjected to the standard Kolomogorov-Smirnov distribution null hypothesis test. The test was performed with the *kstest* function of MATLAB 2018a (R), resulting in *h* = 0 (acceptance) with a *p*-value = 0.994 of the hypothesis test.

**Figure 4:**
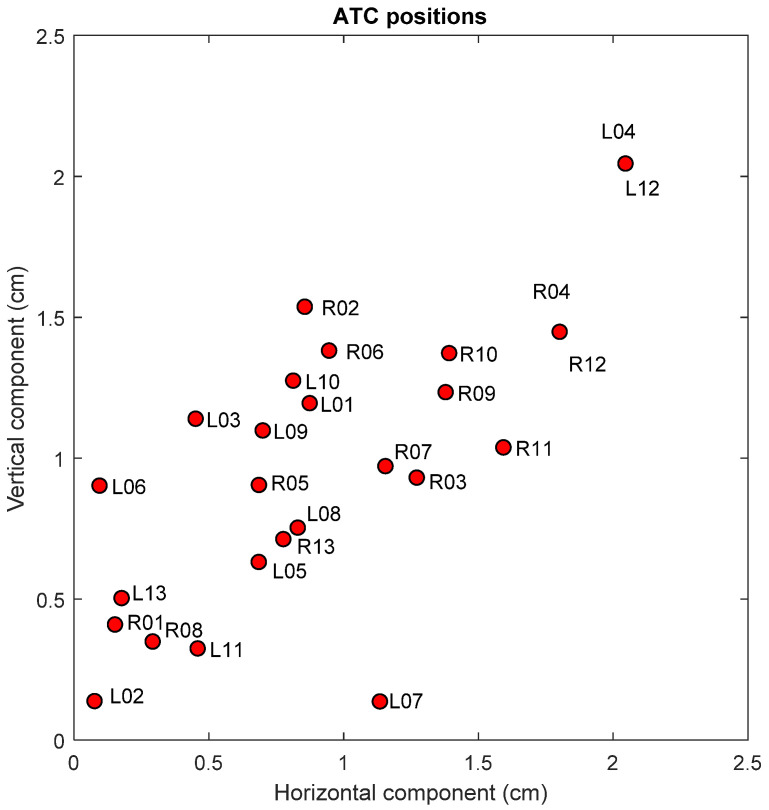
Horizontal and vertical components of the resulting ATC positions for each participant.

**Figure 5:**
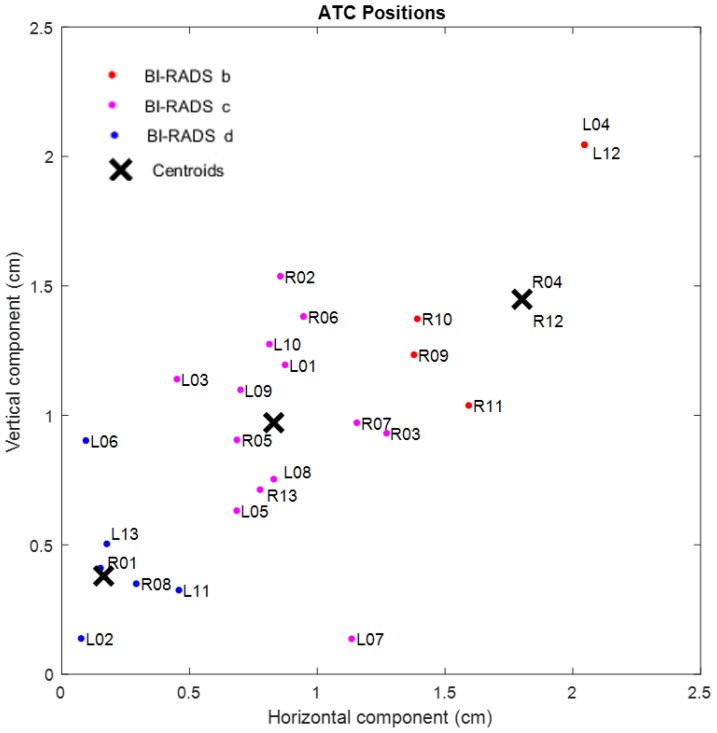
Achieved results applying *K-means* clustering.

A classification of the ATC resulting positions based on the minimum distance to a cluster centroid is proposed. Hence, the radius of the circle generated by the ATC resulted position for each participant’s breast is proposed as the classification parameter. The obtained ATC positions corresponding to all the participants, expressed as the absolute value of the vertical and horizontal components from the breast center, are shown in Figure 4, where *RN* and *LN* represent the right and left resulting ATC position for the *N^TH^* participant, respectively. A *K-means* clustering with the horizontal and vertical components of the obtained ATC positions as input variables was employed. A three-cluster classification with their corresponding centroids is shown in Figure 5.

Considering the assumption that the higher the PBD value, the smaller the radius of the resulting ATC, Figure 3, the BI-RADS PBD obtained with the proposed clustering for each participant is presented in Table 4. Those resulting ATC positions within one cluster and tending to another one are denoted by a hyphen. For instance, the resulting ATC position for R03 is within the cluster corresponding to BI-RADS c and tends to the one corresponding to BI-RADS b; therefore, the resulting BI-RADS is denoted as c-b.

## 4. Discussion

The three defined classification clusters applying *K-means* clustering method to the obtained ATC positions for all the participants are depicted in Figure 5. A relationship between the BI-RADS PBD given by the mammary radiology expert and the resulting ATC clustering is observed. Most of the BI-RADS PBD cases resulted in the three defined classification clusters in a distinguishable way.

It is possible to observe from the resulting centroid position of each cluster that the closer its distance to the center, the larger the breast density. In this way, the centroid for a BI-RADS PBD a would be located even further than the corresponding to BI-RADS b. 

According to the PBD results evaluated by the specialist and the described clustering outcomes, Table 3 and Table 4, only 42.2% of the cases completely agree. However, considering the trend of the resulting ATC positions, the coincidence is 69.2%. One aspect to take into account is that the subjective evaluation carried out by the specialist strongly depends on his experience. Therefore, it is possible to consider that the proposed clustering outcome does not agree with the one obtained by the specialist when there is a completely opposite classification result. According to the above, a result that does not agree is when the clustering outcome is BI-RADS b and the specialist evaluation is BI-RADS d, and vice versa. Considering this criterion, it can be observed that only cases R8 and L11 do not agree. 

**Figure 6:**
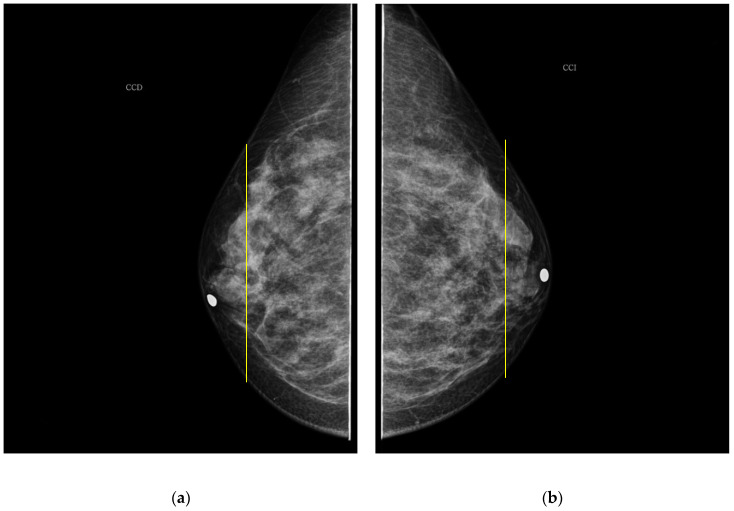
CC mammogram images of participant 9 and relative electrode ring position (yellow vertical line): (**a**) right breast; (**b**) left breast.

As it is noted, the same BI-RADS PBD for both breasts in all the participants was obtained by the specialist; meanwhile, a 61.5% was achieved by the described ATC clustering. An important uncertainty factor to be considered is that the electrodes ring position in most of the cases is not exactly the same on both breasts for each participant. As a consequence, different bioimpedance values are measured because the breast tissue distribution is not the same. For instance, the specialist evaluation for both breasts in participant 9 is BI-RADS c, while the described ATC classification resulted in BI-RADS b-c and c for the right and left breast, respectively. Such difference may be explained by analyzing the relative electrode ring positions on both breasts. The CC mammogram images of both breasts are shown in Figure 6, where the electrodes ring position is illustrated with a yellow vertical line. As it is observed, the left breast has less adipose tissue than the right breast. This amount of fatty tissue is the reason why a higher bioimpedance value is measured.

**Figure 7:**
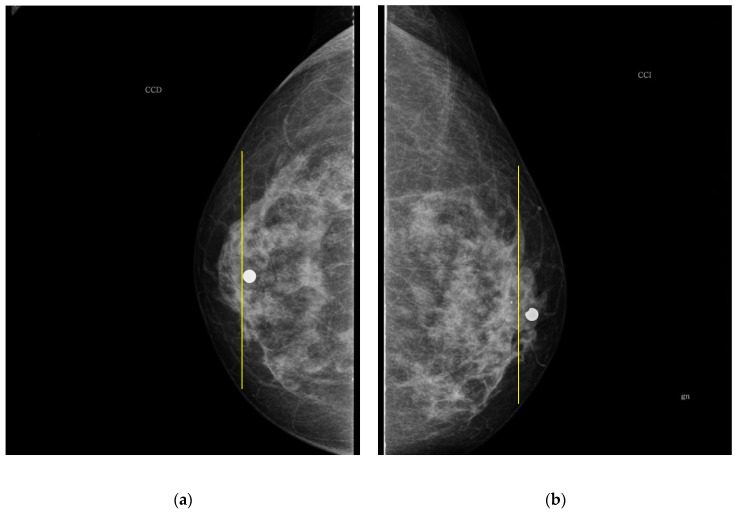
CC mammogram images of participant 1 and relative electrode ring position (yellow vertical line): (**a**) right breast; (**b**) left breast.

The PBD evaluated by the specialist for participant 1 is BI-RADS c for both breasts, and the outcome for the described clustering method is BI-RADS d and c for right and left breast, respectively. The CC mammography images for both breasts are presented in Figure 7, where the respective electrode ring positions are illustrated by the vertical yellow line. As it can be observed, there is a greater breast tissue in the plane covering the vertical yellow line of right breast than that corresponding to the left one. That is the reason why the resulting sectional PBD of right breast is greater than the left breast.

**Figure 8:**
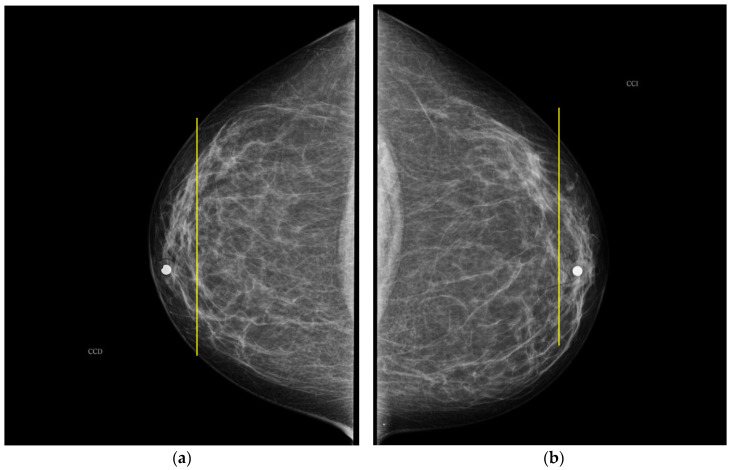
CC mammogram images of participant 5 and relative electrode ring position (yellow vertical line): (**a**) right breast; (**b**) left breast.

In the case of participant 5, both breasts are BI-RADS b and c according to the specialist and the ATC classification algorithm, respectively. Such resulting greater PBD is due to the presence of large mammary gland covered by the measurement plane of the electrode ring in both breasts, as shown in Figure 8.

**Figure 9:**
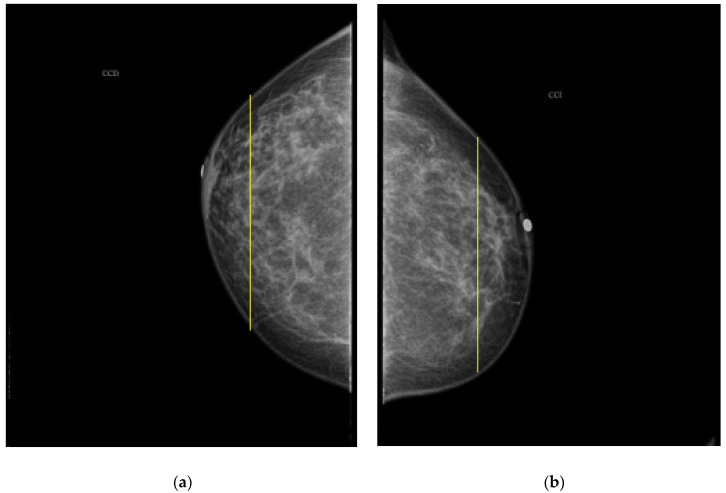
CC mammogram images of participant 4 and relative electrode ring position (yellow vertical line): (**a**) right breast; (**b**) left breast.

**Figure 10:**
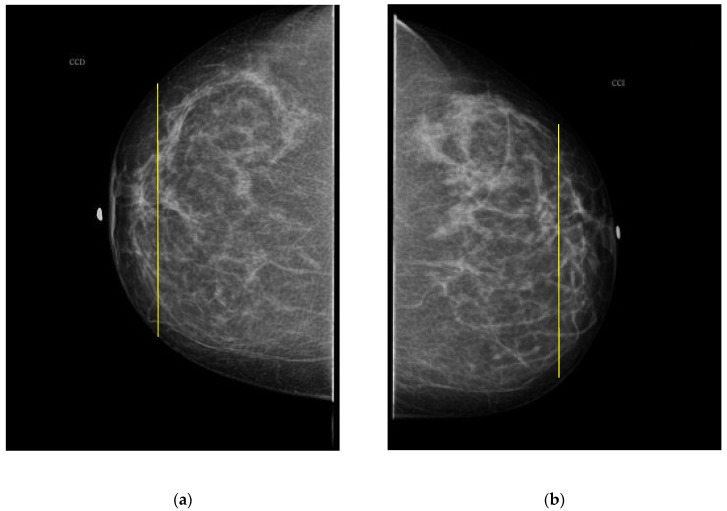
CC mammogram images of participant 12 and relative electrode ring position (yellow vertical line): (**a**) right breast; (**b**) left breast.

As it is observed from the obtained results, the ATC algorithm PBD outcomes agree with the ones diagnosed by the mammary radiology specialist when a representative homogeneity zone is covered by the electrodes ring plane for breasts having homogenous breast tissue distribution (HBTD). This is the case for both breasts of participants 4 and 12, whose CC mammograms images are shown in Figure 9 and Figure 10, respectively.

It is important to note that a woman’s BI-RADS classification may change over time. According to studies by US radiologists, recategorization of sequential screening examinations, e.g., from dense (BI-RADS c or d) to not dense (BI-RADS a or b) or vice versa, occurs in approximately 13–19% of women [30,31]. However, such reclassification of the mammary density status from year to year makes the assessment of women’s breast cancer risk difficult, as well as the informed screening and care decisions [31]. On the other hand, routine PBD measurement is highly recommended, because this marker has high potential in research on etiology and prevention of breast cancer [2]. The described proposal method would be suitable for PBD measuring in a routine way, due to its simple application procedure and most import to the fact that it does not involve radiant energy exposure. The most widely used methods for breast density evaluation are subjective, since they depend on the mammary radiology specialist appreciation and experience. Although the use of a computational system for PBD evaluation has high reliability, a setting of a dichotomous threshold between dense and non-dense tissue is required [32]. In addition, a gradual transition between tissues is not allowed, and image processing variations are not considered in such systems, for instance the intensity change between neighboring image pixels [14].

## 5. Conclusions

A new breast density evaluation method using the ATC algorithm, which is based on breast bioimpedance measurements for locating minimum bioimpedance breast zones, is described in this paper. The proposed methodology was applied to thirteen female participants in one case study. The obtained ATC positions were classified according to the closest distance to a cluster centroid, where three clusters were defined. The clustering outcomes for each participant were compared with the BI-RADS breast density given by an experienced mammary radiologist specialist. The three defined clusters in the described ATC position clustering agree in most of the cases with the three BI-RADS density found by the mammary radiologist specialist. The general trend was the higher the BI-RADS breast density, the smaller the resulting ATC radius and vice versa.

The BI-RADS breast density defined by the described method depends on the breast tissue covered by the plane of the measurement electrodes ring. The electrode ring position in some cases does not cover a representative area of the global breast density. In such cases, the described method outcomes do not agree with the BI-RADS classification given by the global breast density evaluation carried out by the mammogram radiologist expert. Hence, it is possible to state that the BI-RADS density classification with the described ACT clustering method depends on the relative electrode ring position on the breast and on the woman’s global mammary gland distribution. One potential solution to this issue is to increase the bioimpedance measurement area covering more breast tissue in order to achieve a global BI-RADS classification. Currently, the authors are researching the described methodology using a greater number of measurement electrode ring arrays (at least two) in order to achieve a breast bioimpedance measurement in parallel transverse breast areas, expecting in this way a greater precision in the determination of the breast density percentage.

None of the participants felt discomfort or pain during the bioimpedance measurement procedure, unlike the breast pain or discomfort suffered throughout a mammogram procedure.

The described breast density method is a promising approach that might be applied as an auxiliary tool to the mammography in order to obtain more precise and objective results for evaluation of BI-RADS breast density and potential breast cancer risk. The proposed methodology is suitable to be implemented as a portable and minimally invasive solution applicable to women of any age because it does not imply radiation exposure. 

## Figures and Tables

**Table 1:** BI-RADS classification and its relationship with mammography.

BI-RADS PBD Classification	Mammography			
	Right (CCR)	Left (CCL)	Right (MLOR)	Left (MLOL)
a = 0–25% (Homogeneous adipose)	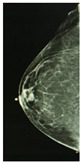	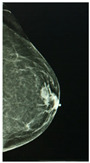	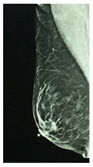	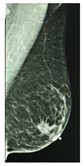
b = 25–50% (Heterogeneous dispersed)	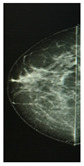	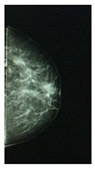	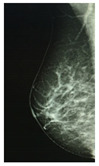	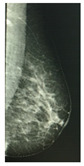
c = 50–75% (Dense heterogeneous)	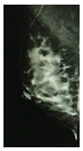	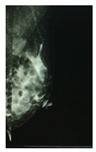	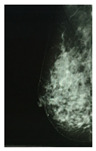	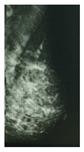
d = 75–100% (Extremely dense)	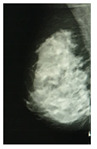	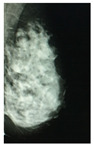	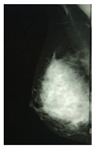	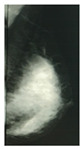

**Table 2:** PBD evaluated by mammary radiology specialist.

Participant	BI-RADS Classification		Participant	BI-RADS Classification	
	Right	Left		Right	Left
1	c	c	8	b	b
2	c	c	9	c	c
3	c	c	10	c	c
4	b	b	11	b	b
5	b	b	12	b	b
6	c	c	13	d	d
7	b	b	-	-	-

**Table 3:** ATC results and CC mammography images of each participant.

ATC Results	Mammography Images
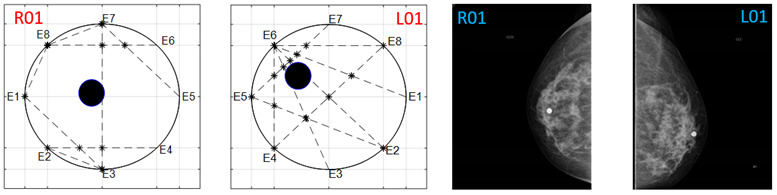	
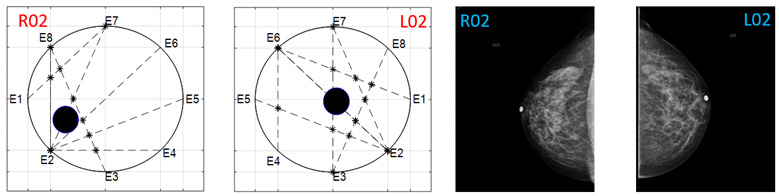	
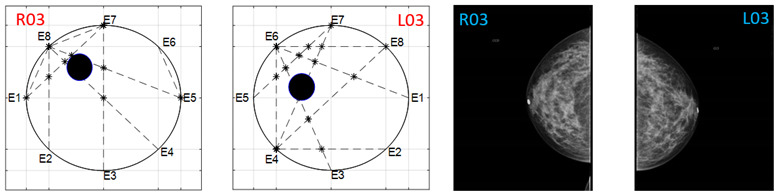	
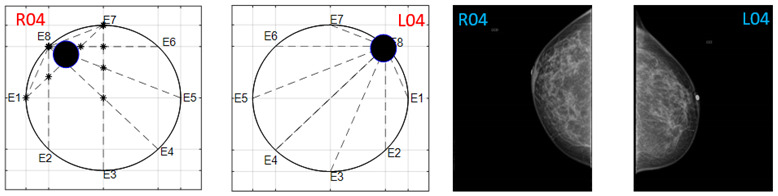	
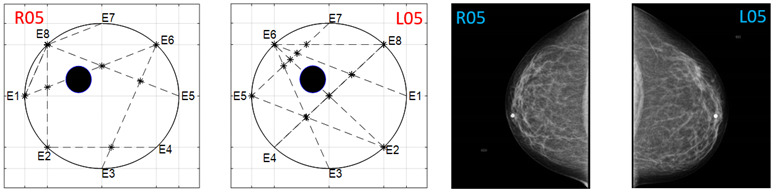	
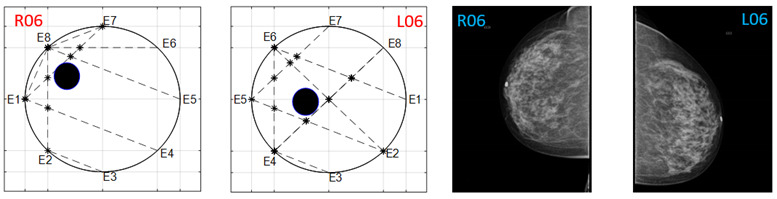	
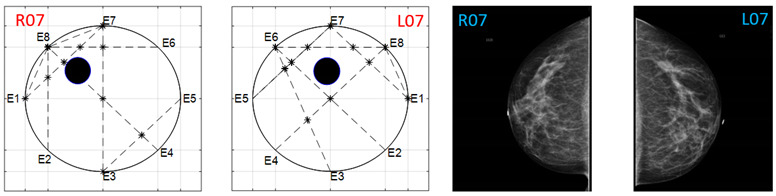	
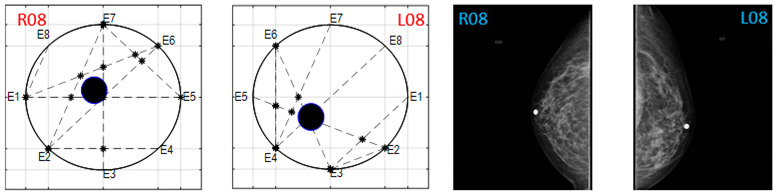	
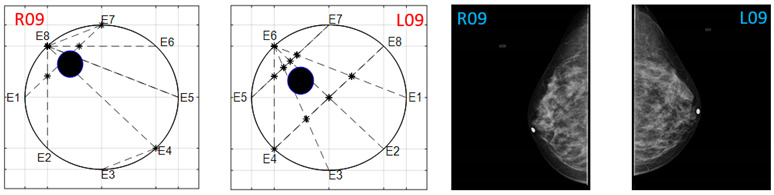	
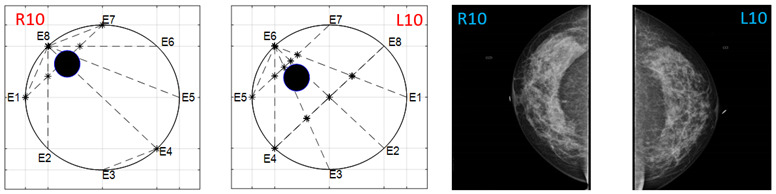	
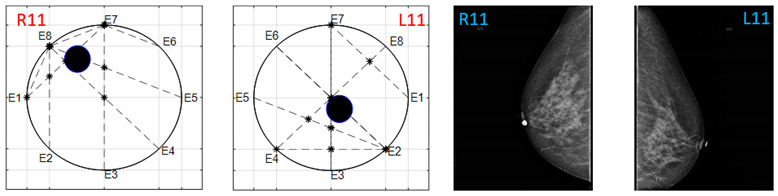	
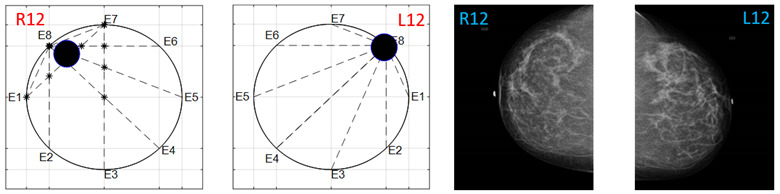	
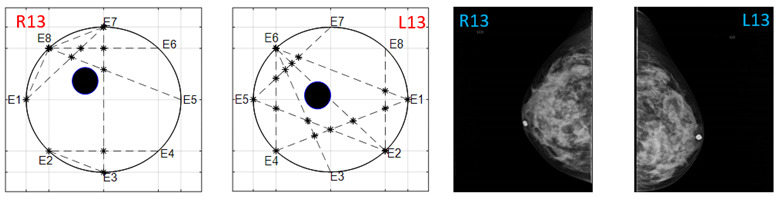	

**Table 4:** BI-RADS PBD achieved with proposed clustering.

Participant	Resulted BI-RADS PBD		Participant	Resulted BI-RADS PBD	
	Right	Left		Right	Left
1	d	c	8	d	c
2	c	d	9	b-c	c
3	c-b	c-d	10	b-c	c
4	b	b	11	b	d
5	c	c	12	b	b
6	c	d-c	13	c	d
7	c-b	c-b	-	-	-

## Data Availability

The Matlab code of the developed Anomaly Tracking Circle algorithm, the mammograms as well as the measured electrical impedance magnitude and phase data for the thirteen participants described in this paper are available in the following link: https://drive.google.com/drive/folders/1MWe6ca4Rk8MoKoOuoHqWGwA8audF-ORF?usp=sharing, (accessed on 6 February 2022).
